# Calibration and Noise Identification of a Rolling Shutter Camera and a Low-Cost Inertial Measurement Unit

**DOI:** 10.3390/s18072345

**Published:** 2018-07-19

**Authors:** Chang-Ryeol Lee, Ju Hong Yoon, Kuk-Jin Yoon

**Affiliations:** 1 School of Electrical Engineering and Computer Science, Gwangju Institute of Science and Technology (GIST), Gwangju 61005, Korea; crlee@gist.ac.kr; 2 Korea Electronics Technology Institute (KETI), Seongnam-si 13509, Korea; jhyoon@keti.re.kr; 3 Department of Mechanical Engineering, Korea Advanced Institute of Science and Technology (KAIST), Daejeon 34141, Korea

**Keywords:** heterogeneous sensor calibration, system identification, visual-inertial navigation

## Abstract

A low-cost inertial measurement unit (IMU) and a rolling shutter camera form a conventional device configuration for localization of a mobile platform due to their complementary properties and low costs. This paper proposes a new calibration method that jointly estimates calibration and noise parameters of the low-cost IMU and the rolling shutter camera for effective sensor fusion in which accurate sensor calibration is very critical. Based on the graybox system identification, the proposed method estimates unknown noise density so that we can minimize calibration error and its covariance by using the unscented Kalman filter. Then, we refine the estimated calibration parameters with the estimated noise density in batch manner. Experimental results on synthetic and real data demonstrate the accuracy and stability of the proposed method and show that the proposed method provides consistent results even with unknown noise density of the IMU. Furthermore, a real experiment using a commercial smartphone validates the performance of the proposed calibration method in off-the-shelf devices.

## 1. Introduction

An inertial measurement unit (IMU) and a camera form a widely used sensor configuration for mobile platform localization. For example, visual-inertial SLAM [1,2,3,4,5,6] is an alternative navigation method in GPS-denied environments such as tunnels and indoor areas where GPS signals are not available. In particular, a MEMS-based IMU and a rolling shutter camera, which capture images line-by-line, are commonly used owing to their low-cost sensing capability.

To use inertial measurements and images simultaneously, calibration of these two heterogeneous sensors is very critical. The calibration of a low-cost IMU and a rolling shutter camera has been studied for last decades in robotics and computer vision community [7,8,9,10,11,12,13,14,15,16,17,18,19]. Previous studies [7,8,9,10,11,12,13,14,15] have separately estimated the IMU intrinsics (i.e., bias, scale factor, and misalignment) and rolling shutter camera intrinsics (i.e., focal length, principle point, and rolling shutter readout time), and then their extrinsics (i.e., relative rotation and translation and time delay) are obtained using the given intrinsic parameters. However, separate calibration is a cumbersome and time-consuming task because each calibration has different procedures. Moreover, intrinsic calibration errors are negatively propagated to extrinsic parameters, which are estimated using pre-computed intrinsics. For these reasons, joint intrinsic and extrinsic calibration of the IMU and the camera has been proposed [16,17,18,19]. These approaches simultaneously minimize the calibration errors of intrinsics and extrinsics.

Most IMU–camera calibration methods estimate the calibration parameters with fixed noise densities of the IMU and camera measurements, which are typically handled as tuning parameters. To achieve accurate calibration, we need to know such noise densities of the IMU and camera. Fortunately, for cameras, the variation in noise densities is not large because calibration patterns (e.g., a checkerboard) are used for corner extraction. Therefore, it is dependent on feature extraction algorithms, whose accuracy in terms of pixels can be easily evaluated and is well-known [20]. In contrast, setting the IMU noise density parameters is difficult and heuristic, because IMUs have various noise densities according to the types and costs of IMUs, and the noise density is not intuitive. Furthermore, noise information of low-cost IMUs is not provided in general.

To address these problems, we jointly estimate the noise density of the low-cost IMU and the intrinsic/extrinsic calibration parameters through the graybox system identification, which estimates the unknown parameters describing the prediction of the dynamic system well [21,22,23]. In general, the graybox method is implemented on a nonlinear optimization problem that minimizes the residual between the predicted and observed measurements of filtering (i.e., the Kalman filter). The proposed framework is composed of two types of graybox methods, which include filtering and optimization, for calibration and noise identification, respectively.

For calibration, rather than finding the calibration parameters in a single optimization step, we divide the calibration process into the filtering step for initialization and the optimization step for refinement. As a result, the convergence time of our framework becomes much faster than the single optimization method. This is because it finds sub-optimal calibration parameters in the filtering step, whose computation time is more efficient than the optimization, and then uses the parameters as an initial value of optimization. Hence, the good initial estimates from filtering reduces the convergence time of optimization for the calibration problem. In fact, it is difficult to design both the calibration and noise parameter estimation problems together in the single optimization step. In the filtering step, we utilize the unscented Kalman filter (UKF) to address the strong nonlinearity of our calibration problem, and its state vector consists of the intrinsic/extrinsic calibration parameters as well as the IMU motion parameters including the position, velocity, and orientation. In the optimization step, we refine the calibration parameters with the noise density estimated from the previous filtering step, while the UKF only estimates the IMU motion including the position, velocity, and orientation.

For noise identification, the optimizer estimates the noise density that attempts to make the UKF converge properly while enabling the estimation of calibration parameters by UKF. Unlike the previous graybox method [23], we define both the prediction errors and state error covariances, which is a constraint, as the cost function of optimization. The IMU noise density is parameterized in terms of system noise covariances in UKF, and both the prediction errors and state error covariances in the cost function involve the system noise covariance in each frame. Without the constraint, the optimization solver forces the system noise covariance to become very large, because it estimates the noise density, which is over-fitted to measurements minimizing the errors (i.e., residuals) in UKF. To avoid such divergence, the proposed method not only minimizes the residuals but also constrains the state error covariances. The initialization of the noise parameters for this optimization is performed using a grid search strategy with the cost function for noise estimation.

**Figure 1:**
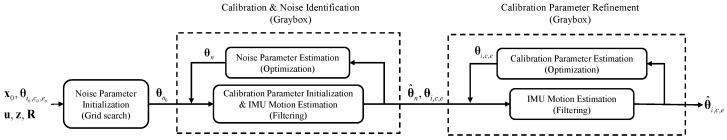
Overall framework of the proposed algorithm. $x_{0}$ is the initial state vector and $\theta_{i,c,e}$ indicates IMU intrinsics (*i*), camera intrinsics (*c*), and extrinsics (*e*). u and z denote inertial and visual measurements, respectively. R is a known measurement noise covariance. The notation $\hat{\cdot }$ denotes the final estimates and subscript ${}_{0}$ denotes an initial step.

We demonstrate the overall procedure of the proposed method with the input and output in Figure 1, and summarize the outputs as follows.

- IMU intrinsics: biases, scale factors, and misalignments
- Camera intrinsics: focal length, radial distortion coefficients, and rolling shutter readout time
- IMU–camera extrinsics: relative rotation, translation, and time delay between an IMU and a camera
- IMU noise parameters: noise density of a gyroscope and an accelerometer

The remainder of this paper is organized as follows. Section 2 briefly reviews the previous studies related to IMU–camera calibration. Section 3 illustrates the problem formulation for our calibration method. A detailed description about the calibration system model and its measurement model is provided in Section 4. Section 5 describes the framework and the implementation of our calibration method. Section 6 analyzes the experimental results to validate the advantages of the proposed calibration method. Finally, we conclude the paper in Section 7.

## 2. Related Work

As mentioned in Section 1, the goal of calibration is to obtain the intrinsic parameters of an IMU and a camera and extrinsic parameters of these two sensors. In this section, we review the separate calibration methods of each sensor and the joint calibration methods of an IMU and a camera. In addition, we introduce several studies related to the noise identification of an IMU.

### 2.1. Separate Calibration

The intrinsic calibration of a camera is a well-known problem in computer vision [24]. Therefore, rather than reviewing conventional camera intrinsic calibration methods, we focus on rolling shutter calibration, which additionally finds the readout time delay between lines. Geyer et al. [10] proposed the exploitation of LED flashing at a high frequency to estimate the delay. They assumed constant velocity models and removed the lens for good illumination. In contrast, Ringaby et al. [11] introduced a more efficient approach that does not need to remove lens. O’Sullivan and Corke [25] designed a new rolling shutter camera model, which can handle arbitrary camera motions and projection models such as fisheye or panoramic model. Oth et al. [12] used video sequences with a known calibration pattern. They used a continuous-time trajectory model with a rolling shutter camera model, and applied a batch optimization approach to estimate the sequential camera poses and the line delay of the rolling shutter camera.

The intrinsic calibration of an IMU has been performed using mechanical platforms such as robotic manipulators, which precisely moves IMU along known motion trajectories [7,8,9]. The intrinsic parameters of an accelerometer and a gyroscope are estimated by comparing the output of IMU and the motion dynamics, i.e., acceleration and angular velocity, which represent the generated known motion. In [26,27], authors proposed to exploit a marker-based motion tracking system or GPS measurements rather than using the expensive mechanical platforms. Recently, calibration methods that do not require any additional device have also been proposed in [13,28].

The extrinsic calibration of an IMU and a camera has been studied for the last decade in robotics community. Mirzaei and Roumeliotis [14] first presented a filter-based IMU–camera calibration method. They exploited the extended Kalman filter (EKF) to approximate nonlinear system and measurement models. Hol and Gustafsson [23] adopted the graybox system identification with which they integrated a filter-based framework and an optimization method. Kelly and Sukhatme [2] employed UKF to handle the nonlinear calibration model. They also proposed a general self-calibration algorithm that does not require additional equipment. Unlike other approaches, they included gravity as one of the state parameters to be estimated considering geo-location dependency. Dong–Si and Mourikis formulated rotation calibration as a convex problem [29]. Fleps et al. [30] proposed a new cont function that includes alignment between each trajectory of an IMU and a camera. Furgale et al. [15] proposed spatial and temporal calibration by modeling the IMU and camera motion based on a spline function. In photogrammetry community, the calibration of multiple heterogeneous sensors is called bore-sight alignment. Blazquez and Colomina [31] utilized INS/GNSS and cameras on unmanned aerial vehicle (UAV), and estimated relative boresight (rotation) and lever-arm (translation) between these sensors. Cucci et al. [32] proposed self-calibration of the sensors and 3D pose estimation using a low-cost IMU, GNSS, and cameras in a small UAV.

### 2.2. Joint Calibration

Joint estimation of the intrinsic and extrinsic parameters of an IMU and a camera is efficient because it does not need to conduct calibration independently for each sensor. Furthermore, it generates more accurate results because it minimizes the intrinsic and extrinsic calibration errors simultaneously. Moreover, the joint estimation is more practical since most mobile devices are equipped with a low-cost IMU and a rolling shutter camera. The joint estimation has been introduced in [16], where a low-cost IMU and a pinhole camera model were used, and in [33], where a low-cost gyroscope and a rolling shutter camera were used. Li et al. additionally considered the temporal synchronization between the IMU and the camera and the rolling shutter camera model for calibration of a low-cost IMU and a rolling shutter camera [34,35]. Lee et al. proposed to suppress uncertain noises of low-cost IMU for IMU–camera calibration [18]. Rehder et al. proposed the calibration of multiple IMUs and a camera, and considered the transformation between individual axes of the IMUs [19]. Li et al. introduced self-calibration methods for a low-cost IMU–camera system while estimating sequential camera poses in vision-aided inertial navigation [17]. It estimates the intrinsic parameters, extrinsic parameters, rolling shutter readout time, and temporal offset between an IMU and camera measurements. We highlight the differences between the proposed method and the aforementioned methods in Table 1.

### 2.3. Noise Identification

The Allan variance (AV) and the non-parametric power spectral density are the classical tools used to describe noise characteristics of an IMU. Refer to [36] for a detailed description of these procedures. Vaccaro et al., introduced the automatic noise identification by computing and matching the covariances of the AV [37]. In addition, the IMU noise parameter estimation methods were proposed in [38,39] by using a maximum likelihood with the KF in both online and offline manners.

## 3. Problem Formulation

**Figure 2:**
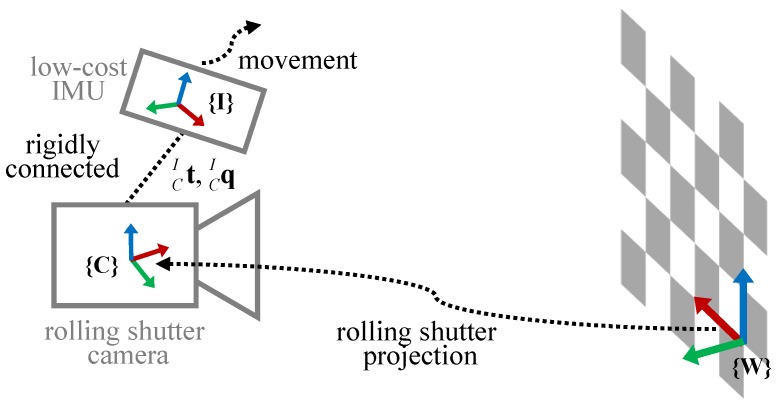
Schematic overview and the calibration coordinate description for the rigidly connected low-cost IMU and rolling shutter camera. The notations ({I}, {C}, and {W}) denote the coordinates of the IMU, the rolling shutter camera, and the world, respectively.

Our system comprises a low-cost IMU and a rolling shutter camera, and these two sensors are rigidly connected as in Figure 2. The IMU measures 3-DOF acceleration and 3-DOF angular velocity, and a rolling shutter camera captures an image row-by-row. In this paper, we represent the coordinates of the IMU, the camera, and the world by {I}, {C}, and {W}, respectively. These notations are used as a left superscript to denote a reference coordinate and a left subscript to represent a target coordinate, as shown in Figure 2. For example, the 3D position p of the IMU coordinate {I} from the world coordinate {W} is represented by ${}_{I}^{W}p$, and the 3D translation t of the camera {C} coordinate from the IMU coordinate {I} is denoted by ${}_{C}^{I}t$.

To generate calibration data, we sequentially obtain inertial measurements and images by capturing a checkerboard along three-axis linear and angular motion. The inertial measurements u from the IMU are composed of acceleration $a\in R^{3}$ and angular velocity $w\in R^{3}$. Visual measurements $z\in R^{2\times M}$ comprise *M* corners of the checkerboard in the image coordinate. Given the visual and inertial measurements, we formulate the calibration and noise identification as a minimization problem as follows.

(1) $$\hat{\theta }=\min_{\theta }E(\theta ;\;u,z),$$

where the cost function E(·) illustrates the alignment error between motions of the IMU and the camera, which is computed from the visual and inertial measurements u,z, with the calibration and noise parameters denoted by $\theta =\{\theta_{i},\theta_{c},\theta_{e},\theta_{n}\}$. The intrinsic parameter $\theta_{i}$ of the IMU describes the inherent difference between the raw measurements of sensors and the real-world value. The intrinsic parameter $\theta_{c}$ of the rolling shutter camera explains the mapping from the camera coordinate to the image coordinate. The extrinsic parameter $\theta_{e}$ between the IMU and the camera describes the relative geometric difference between the IMU coordinate and the camera coordinate. The noise parameter $\theta_{n}$ of the IMU represents noise characteristics of inertial measurements.

(2) $$\left\{\begin{array}{lll} u^{\prime } & =a(u,\theta_{i}) & :\mathrm{IMU}\;\mathrm{intrinsics}\\ z & =b{(}^{C}X,\theta_{c}) & :\mathrm{Camera}\;\mathrm{intrinsics}\\ {}^{I}X & =c{(}^{C}X,\theta_{e}) & :\text{IMU-camera}\;\mathrm{extrinsics}\\ \theta_{n} & =d(u) & :\mathrm{IMU}\;\mathrm{noises} \end{array}\right.$$

where $u^{\prime }$ indicates the intrinsically calibrated inertial measurements, and $X^{C}\in R^{3}$ indicates the position of a three-dimensional point in a camera coordinate. a(·),b(·),c(·), and d(·) are functions for describing each parameter. The calibration and noise parameters are composed of several variables. Table 2 shows a detailed description of the unknown variables in each parameter.

## 4. Model

In this section, we describe the system and measurement models for calibration as follows.

(3) $$\begin{array}{c} x_{k}=F\left(x_{k-1},u_{k}-1\right)+w_{k-1}\\ z_{k}=H\left(x_{k}\right)+v_{k}, \end{array}$$

where the state vector x represents the sensor motion state and calibration parameters, and are propagated with the inertial measurements u based on the system model $F\left(\cdot \right)$. The nonlinear measurement model $H\left(\cdot \right)$ describes the relation between the state vector and the measurement z. w is a process noise and v is a measurement noise. Both are assumed to be white Gaussian noise, w∼N(0,Q) and v∼N(0,G) with the noise covariance Q and G, respectively. Subscripts k−1 and *k* denote time steps. We explain the aforementioned notations in detail as follows.

We include the sensor motion parameters in the state vector. Therefore, the state vector $x\in R^{40}$ is composed of a motion state ρ, extrinsic parameters $\theta_{e}$, IMU intrinsic parameters $\theta_{i}$, and camera intrinsic parameters $\theta_{c}$.

(4) $$x={\left[\begin{array}{cccc} \rho^{⊤} & \theta_{e}^{⊤} & \theta_{i}^{⊤} & \theta_{c}^{⊤} \end{array}\right]}^{⊤}.$$

Here, the motion state $\rho \in R^{10}$ is represented by

(5) $$\rho ={\left[\begin{array}{ccc} {}_{I}^{W}p^{⊤} & {}_{I}^{W}v^{⊤} & {}_{I}^{W}q^{⊤} \end{array}\right]}^{⊤},$$

where ${}_{I}^{W}p\in R^{3}$ and ${}_{I}^{W}v\in R^{3}$ indicate the position and velocity of the IMU coordinate from the world coordinate, respectively; and ${}_{I}^{W}q\in R^{4}$ is an orientation expressed as a unit quaternion of the IMU coordinate from the world coordinate.

The extrinsic parameter $\theta_{e}\in R^{8}$ is denoted by

(6) $$\theta_{e}={\left[\begin{array}{ccc} {}_{I}^{C}t^{⊤} & {}_{I}^{C}q^{⊤} & \lambda_{d} \end{array}\right]}^{⊤},$$

where ${}_{I}^{C}t\in R^{3}$ is a translation from the IMU coordinate to the camera coordinate, and ${}_{I}^{C}q\in R^{4}$ is a rotation represented as a unit quaternion from the IMU coordinate to the camera coordinate. $\lambda_{d}$ is a temporal delay between the IMU and the camera.

The IMU intrinsic parameter $\theta_{i}\in R^{18}$ is represented by

(7) $$\theta_{i}={\left[\begin{array}{cccccc} b_{a}^{⊤} & b_{g}^{⊤} & m_{a}^{⊤} & m_{g}^{⊤} & s_{a}^{⊤} & s_{g}^{⊤} \end{array}\right]}^{⊤},$$

where $b_{a}\in R^{3}$, $m_{a}\in R^{3}$, and $s_{a}\in R^{3}$ indicate a bias, a misalignment, and a scale factor of the accelerometer, respectively. $b_{g}\in R^{3}$, $m_{g}\in R^{3}$, and $s_{g}\in R^{3}$ are the parameters of the gyroscope.

The camera’s intrinsic parameter $\theta_{c}\in R^{4}$ is represented by

(8) $$\theta_{c}={\left[\begin{array}{ccc} f & d & \lambda_{rs} \end{array}\right]}^{⊤},$$

where f∈R is the focal length, $d\in R^{2}$ is radial distortion, and $\lambda_{rs}\in R$ is the rolling shutter readout time of the rolling shutter camera.

### 4.1. System Model: Low-Cost IMU

**Figure 3:**
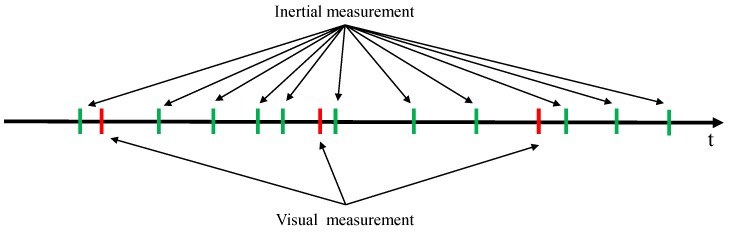
Time diagram for visual and inertial measurements. The green and red vertical lines indicate the timestamps of the IMU and camera measurements, respectively.

We describe the system model F(·) in Equation (3) performing the transition of the state vector x. The visual and inertial measurements are obtained at different frame rates and their time intervals have some variation. An example of this time interval is demonstrated in Figure 3.

The state transition (or prediction) is sequentially performed with the inertial measurements $u_{s}$ until a new visual measurement is obtained, as demonstrated in Figure 3. The number of predictions becomes $\#prediction=\#u_{s}+1$. We define the time interval Δt for each prediction as follows.

(9) $$\Delta t=\left\{\begin{array}{cc} t_{imu,1}-\left(t_{img,k-1}+\lambda_{d}\right) & :\mathrm{First}\\ t_{imu,s}-t_{imu,s-1} & :\mathrm{Middle}\\ \left(t_{img,k}+\lambda_{d}\right)-t_{imu,end} & :\mathrm{Last} \end{array}\right.$$

where *k* is the time-step of the visual measurements, while *s* is that of the inertial measurements.

The nonlinear system model in Equation (3) is defined as follows.

(10) $$\begin{array}{cc} F\left(x_{k-1},u_{s}\right)= & \left[\begin{array}{c} \begin{array}{c} F_{mot}\left(\rho_{k-1},u_{s}\right)\\ \theta_{e,k-1}\\ \theta_{i,k-1}\\ \theta_{c,k-1} \end{array} \end{array}\right]. \end{array}$$

Here, the motion state is sequentially predicted with the IMU measurement $u_{s}$ and the time interval Δ*t*, and the other calibration parameters are considered as constant because they are static and two sensors are rigidly connected. Note that we do not regard the bias states as smoothly time varying parameters, unlike [19] because the input inertial measurements for calibration are obtained for a short time (about 1–2 min).

(11) $$\begin{array}{c} F_{mot}\left(\rho_{k-1},u_{s}\right)=\left[\begin{array}{c} \begin{array}{c} {}_{I}^{W}p_{k-1}+{}_{I}^{W}v_{k-1}\Delta t\\ {}_{I}^{W}v_{k-1}+{}_{I}^{W}a_{s}\Delta t\\ {}_{I}^{W}q_{k-1}+\Omega \left(-w_{s}\frac{\Delta t}{2}\right){}_{I}^{W}q_{k-1} \end{array} \end{array}\right], \end{array}$$

where the quaternion kinematic function $\Omega :R^{3}\to R^{4\times 4}$ and the angular velocity $w_{s}$ explain the time variation of IMU orientation. The linear acceleration ${}_{I}^{W}a_{s}$ and angular velocity $w_{s}$ are converted from the raw measurements $a_{m}$ and $w_{m}$ via the intrinsic parameters of the IMU as follows.

(12) $$\left[\begin{array}{c} {}_{I}^{W}a_{s}\\ w_{s} \end{array}\right]=\left[\begin{array}{c} \begin{array}{c} {}_{I}^{W}R\left(S_{a}M_{a}a_{m}+b_{a}\right)-g\\ S_{g}M_{g}w_{m}+b_{w} \end{array} \end{array}\right],$$

where ${}_{I}^{W}R\in R^{3\times 3}$ is a rotation matrix expressing the orientation of the IMU, which is converted from ${}_{I}^{W}q$, and g is the gravity vector in the world coordinate. The scale factor matrices $S_{a}$ and $S_{g}$ are generated by the scale factor states $s_{a}$ and $s_{g}$ as

$$\begin{array}{cc} S_{a}= & \left[\begin{array}{c} \begin{array}{ccc} s_{a,x} & 0 & 0\\ 0 & s_{a,y} & 0\\ 0 & 0 & s_{a,z} \end{array} \end{array}\right],\;S_{w}=\left[\begin{array}{c} \begin{array}{ccc} s_{g,x} & 0 & 0\\ 0 & s_{g,y} & 0\\ 0 & 0 & s_{g,z} \end{array} \end{array}\right] \end{array}$$

and the misalignment matrices (i.e., $M_{a}$ and $M_{g}$) between the orthogonal IMU coordinate and the accelerometer/gyroscope coordinates are represented as below

$$\begin{array}{cc} M_{a}= & \left[\begin{array}{c} \begin{array}{ccc} 1 & m_{a,x} & m_{a,y}\\ 0 & 1 & m_{a,z}\\ 0 & 0 & 1 \end{array} \end{array}\right],\;M_{g}=\left[\begin{array}{c} \begin{array}{ccc} 1 & m_{g,x} & m_{g,y}\\ 0 & 1 & m_{g,z}\\ 0 & 0 & 1 \end{array} \end{array}\right]. \end{array}$$

The lower triangular elements become zero because we assume that the *x*-axes of the accelerometer and the gyroscope coincide to one of the orthogonal IMU coordinates as in [13,19].

### 4.2. Measurement Model: Rolling Shutter Camera

The measurement model in Equation (3) describes the relation between the state vector x and the measurements z. The visual measurement z is a set of calibration pattern corners obtained from an image. Unlike a global shutter camera, a low-cost rolling shutter camera captures an image row-by-row. The projective geometry of a rolling shutter camera is formulated as follows.

(13) $$\left[\begin{array}{c} u^{{}^{\prime }}\\ v^{{}^{\prime }} \end{array}\right]∼K{\left[\begin{array}{cc} {}_{C_{v}}^{C_{\frac{h}{2}}}R & {}_{C_{v}}^{C_{\frac{h}{2}}}t \end{array}\right]}_{rs}\left[\begin{array}{cc} {}_{W}^{C}R & {}_{W}^{C}t\\ 0 & 1 \end{array}\right]{}^{W}X,$$

where ${}^{W}X\in P^{3}$ denotes a 3D point in the world coordinate. The intrinsic matrix is represented by $K\in R^{3\times 3}$. The notation ∼ denotes normalization of a projected point. The rotation matrix and translation vector of the world coordinate from a camera coordinate are represented by ${}_{W}^{C}R\in \mathrm{SO}(3)$ and ${}_{W}^{C}t\in R^{3}$, respectively. They are computed from our state vector, which is the position ${}_{I}^{W}p$ and orientation ${}_{I}^{W}q$ of the IMU from the world coordinate and transformation ${}_{C}^{I}t$, ${}_{C}^{I}q$ between the IMU and the camera. ${}_{C_{v}}^{C_{\frac{h}{2}}}R$ and ${}_{C_{v}}^{C_{\frac{h}{2}}}t$ denote rolling shutter transformation from the center row $\left(\frac{h}{2}\right)$ to a row (v) of the projected point because we use the center row as the reference row of an image when defining the rolling shutter distortion. Here, *h* is the height of the image. To compute the rolling shutter transformation, we use the Cayley transform model described in [40].

(14) $${}_{C_{v}}^{C_{\frac{v}{2}}}R≃R\left(\left(\frac{h}{2}-v\right)\lambda_{rs}{}^{C}w\right),\;{}_{C_{v}}^{C_{\frac{v}{2}}}t=\left(\frac{h}{2}-v\right)\lambda_{rs}{}^{C}v,$$

where $\lambda_{rs}$ is the rolling shutter readout time included in the state vector and ${}^{C}w$ and ${}^{C}v$ are angular and linear velocities of the camera. The angular and linear velocities are computed from gyroscope measurements and states.

(15) $${}^{C}w={}_{I}^{C}R{}^{I}w_{s},\;{\;}^{C}v≃{}_{W}^{C}R{}_{I}^{W}v_{k|k-1},$$

where ${}_{I}^{C}R$ is the rotation of the camera from the IMU, ${}^{I}w_{s}$ is an intrinsically calibrated angular velocity of the IMU in Equation (12), and ${}_{I}^{W}v_{k|k-1}$ is a predicted velocity of the IMU. We use them at the closest point to the time step $t_{img}+\lambda_{d}$, as follows.

Finally, each corner $z_{i}\in z$ in the visual measurement is obtained by considering radial distortion.

(16) $$z_{i}=\left[\begin{array}{c} u\\ v \end{array}\right]=H\left(x;\;{}^{W}X\right)=\left(1+d_{1}r^{2}+d_{2}r^{4}\right)\left[\begin{array}{c} u^{{}^{\prime }}\\ v^{{}^{\prime }} \end{array}\right],$$

where the subscript *i* is an index of a projected point and $\{d_{1},d_{2}\}\subset d$ is the radial distortion state. To utilize multiple points, we concatenate the corner positions in the image coordinate as follows.

(17) $$\begin{array}{c} z={\left[{z_{1}}^{⊤}\cdots {z_{M}}^{⊤}\right]}^{⊤}, \end{array}$$

where *M* is the number of corner points.

## 5. Proposed Method

Our calibration and noise identification is based on the graybox system identification [22,41], which is a nonlinear optimization method minimizing the residuals of the Kalman filter (KF). Figure 1 demonstrates the overall framework of the proposed method. We first initialize the noise parameters by grid search because we do not have any prior knowledge on the noise. Then, we estimate the calibration and noise parameters with our novel graybox method, whose cost function considers not only residuals but also state error covariances of KF. Here, the noise parameters are estimated by nonlinear optimization, while the UKF estimates the motion state and the calibration parameters together. Finally, in the refinement step, the optimization module estimates the calibration parameters using the conventional graybox-based calibration [23], whose cost function considers only residuals, while the UKF module only estimates the motion state.

We define the cost function in Equation (1) as a square form of the state error covariances of the UKF and the residuals between the predicted and observed measurements.

(18) $$E_{1}\left(\theta_{n}\right)=\sum_{k=1}^{M}{\left(z_{k}-z_{k|k-1}\right)}^{2}+{\left(diag(S_{k|k})\right)}^{2},\;\;S_{k|k}=H^{⊤}P_{k|k}H$$

where diag(·) is the diagonal of a square matrix and *M* is the number of visual measurements. The covariance $S_{k|k}$ is computed in filtering with the updated state error covariance $P_{k|k}$ and a Jacobian matrix of the measurement function H. The computation of the predicted measurements and the state error covariances in UKF is described in Equation (22).

**Figure 4:**
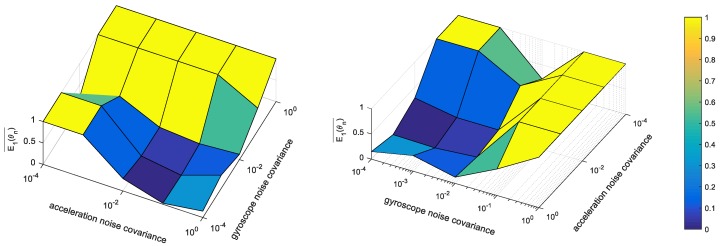
Cost map for noise parameter estimation. $\bar{E_{1}\left(\theta_{n}\right)}$ is the normalized cost function defined in Equation (19).

For the initialization of the noise parameters, we use the same cost function and a grid search strategy. The search range is $0<\theta_{n}<1$, and the grid size is 5 with log-scale intervals. Figure 4 demonstrates the cost map computed by the grid search. Then, we estimate the noise and calibration parameters using the graybox method. For the optimization, we exploit the Levenberg–Marquardt algorithm, which is a nonlinear least square solver.

(19) $$\arg\min_{\theta_{n}}E_{1}\left(\theta_{n}\right)$$

At this time, the calibration parameters are simultaneously estimated by the UKF. Therefore, we estimate the noise parameters that result in the optimal convergence of the UKF for calibration.

### 5.1. Noise and Calibration Parameters in UKF

We adopt the UKF [42] owing to its efficient performance on the nonlinear system and measurement model. The prediction of the state $x_{k-1|k-1}$ and its error covariance $P_{k-1|k-1}$ are formulated as below.

(20) $$\begin{array}{cc} \chi_{k-1|k-1}^{i} & =sp(x_{k-1|k-1},P_{k-1|k-1},Q\left(\theta_{n}\right))\\ \chi_{k|k-1}^{i} & =F(\chi_{k-1|k-1}^{i},u_{k-1},\theta_{i})\\ x_{k|k-1} & =\sum_{i}W_{s}^{i}\chi_{k|k-1}^{i}\\ P_{k|k-1} & =\sum_{i}W_{c}^{i}\left[\chi_{k|k-1}^{i}-x_{k|k-1}\right]{\left[\chi_{k|k-1}^{i}-x_{k|k-1}\right]}^{⊤} \end{array}$$

where sp is the sigma point generation function, χ is the generated sigma points of the state, the superscript *i* is the index of the sigma point (i=1,⋯,N), and $W_{s},W_{c}$ are weights of sigma points for the state and covariance, respectively. The sigma points $\chi_{k-1|k-1}^{i}$ are generated with the state $x_{k-1|k-1}$, the state error covariance $P_{k-1|k-1}$, and the system noise covariance Q. Each sigma point is predicted using system model F(·) with the inertial measurement $u_{k-1}$ and the IMU intrinsic parameter $\theta_{i}$. The system noise covariance $Q_{\rho }$ for motion states is defined from the IMU noise parameter $\left\{\sigma_{a},\sigma_{g}\right\}\subset \theta_{n}$. The system noise covariances for other states are set to zero because they are static. As mentioned before, we regard the bias states as constant parameters, unlike [19].

(21) $$Q_{\rho }=\left[\begin{array}{ccc} {\left(\sigma_{a}{\Delta t}^{2}\right)}^{2}I_{3} & 0_{3} & 0_{3\times 4}\\ 0_{3} & {\left(\sigma_{a}\Delta t\right)}^{2}I_{3} & 0_{3\times 4}\\ 0_{4\times 3} & 0_{4\times 3} & {\left(\sigma_{g}\Delta t\right)}^{2}I_{4} \end{array}\right]$$

The update of the predicted state $x_{k|k-1}$ and the predicted state error covariance $P_{k|k-1}$ are formulated as follows.

(22) $$\begin{array}{cc} \chi_{k|k-1}^{i} & =sp(x_{k|k-1},P_{k|k-1},R)\\ \gamma_{k|k-1}^{i} & =H(\chi_{k|k-1}^{i},\theta_{c},\theta_{e})\\ z_{k|k-1} & =\sum_{i}W_{s}^{i}\gamma_{k|k-1}^{i}\\ P_{z_{k}z_{k}} & =\sum_{i}W_{c}^{i}\left[\gamma_{k}^{i}-z_{k|k-1}\right]{\left[\gamma_{k}^{i}-z_{k|k-1}\right]}^{⊤}\\ P_{x_{k}z_{k}} & =\sum_{i}W_{c}^{i}\left[\chi_{k|k-1}^{i}-x_{k|k-1}\right]{\left[\gamma_{k}^{i}-z_{k|k-1}\right]}^{⊤}\\ K_{k} & =P_{x_{k}z_{k}}P_{z_{k}z_{k}}^{-1}\\ x_{k|k} & =x_{k|k-1}+K_{k}\left(z_{k}-z_{k|k-1}\right)\\ P_{k|k} & =P_{k|k-1}-K_{k}P_{k|k-1}K_{k}^{⊤} \end{array}.$$

The predicted sigma point $\chi_{k|k-1}^{i}$ is transformed through the measurement model H(·) with the camera intrinsic parameter $\theta_{c}$ and the IMU–camera extrinsic parameter $\theta_{i}$. Then, the Kalman gain $K_{k}$ is computed with the predicted measurement covariance ${\hat{P}}_{z_{k}z_{k}}$ and state-measurement cross-covariance matrix ${\hat{P}}_{x_{k}z_{k}}$. The predicted state and state error covariance are updated with the Kalman gain. The measurement noise covariance G is defined as a block-diagonal matrix.

(23) $$G=\left[\begin{array}{ccc} \sigma_{z}^{2}I_{2} & 0_{2} & 0_{2}\\ 0_{2} & \ddots & 0_{2}\\ 0_{2} & 0_{2} & \sigma_{z}^{2}I_{2} \end{array}\right]$$

### 5.2. Refinement

We refine the estimated calibration parameters with the conventional graybox method. Here, we only use the residuals between the predicted measurement and the observed measurement in the cost function, and the filter module only estimates the IMU motion state. As a result, the fixed system noise covariance $Q_{\rho }\in R^{10}$, instead of $Q\in R^{40}$, for motion states is used in UKF. However, unlike [23], which estimated only extrinsic parameters, we estimate the intrinsic parameters of the IMU and the camera as well as the extrinsic parameters. Therefore, the cost function in Equation (1) is defined as follows:

(24) $$\arg\min_{\theta_{i,c,e}}E_{2}\left(\theta_{i,c,e}\right),\;\;E_{2}\left(\theta_{i,c,e}\right)=\sum_{k=1}^{N}{\left(z_{k}-z_{k|k-1}\right)}^{2}.$$

Since this optimization is performed in batch manner, unlike calibration parameter estimation by filtering in the previous step, outliers or disturbances such as abrupt motion or illumination changes can be corrected in this step.

### 5.3. UKF Initialization

In this section, we describe the initialization of the states, the state error covariance, and the measurement noise covariance for the UKF. The initial position and orientation of the IMU are computed by transforming the position and the orientation of the camera at the first frame through the initial relative rotation and translation between the IMU and the camera. The velocity of the IMU is initialized to zero. The initial focal length *f* is set to the width of the image. The relative rotation between the IMU and the camera ${}_{I}^{C}q$ is initialized by the angular velocities of the IMU and estimated rotation from the camera [15]. The IMU provides the raw angular velocity measurements. To compute angular velocities of the camera, we estimate the camera orientations with respect to the checkerboard pattern using homography [24]. Then, the angular velocity is obtained from the derivative of the orientation of the camera. The other parameters—the relative translation ${}_{I}^{C}t$ and time delay $\lambda_{d}$ between the IMU and the camera, biases $b_{a},b_{g}$, misalignments $m_{a},m_{g}$, distortion coefficient d, and rolling shutter readout time $\lambda_{rs}$—are initially set to zero, and scale factors $s_{a},s_{g}$ are set to one. The initial state error covariance matrix is empirically set as shown in Table 3. Based on the pixel localization error of the corner extraction algorithm, the standard deviation $\sigma_{z}$ for the measurement noise covariance is set to 1. The gravity g in the world coordinate is set to ${\left[0,\;0,\;9.81\right]}^{⊤}$, and the *z*-axis of the world coordinate is the vertical direction. The initial calibration parameters for the refinement step are obtained from the estimated calibration parameter from the UKF in the calibration and noise identification step.

## 6. Experimental Results

We evaluate the proposed method on the synthetic and real data. The synthetic data contain synthetically generated motion information of an IMU–camera system and corresponding calibration pattern points. Note that the two sensors are rigidly connected. Using the synthetic data, we validate the accuracy and stability of the proposed method by running 100 Monte-Carlo simulations with fixed noise parameters. Furthermore, we compare the calibration results with and without noise identification using random noise parameters to demonstrate the effect of noise estimation. In real-data experiments, we utilize two experimental setups: a self-designed system, which is equipped with two rolling shutter cameras and one low-cost IMU, and a commercial smartphone, which has a rolling shutter camera and a low-cost IMU. The first setup is specially designed to evaluate the extrinsic calibration accuracy. Since the ground truth cannot be obtained for real-data experiments, we indirectly measure the performance based on loop-closing errors between the two cameras and the IMU. With these two setups, we first analyze the performance of the proposed method based on the standard deviation of the estimate. We also compare the proposed method with hand-measure estimates in terms of extrinsics and existing calibration methods in terms of intrinsics and extrinsics for reference. These comparisons show that the proposed method produces comparable results on the camera intrinsic calibration. In addition, the smartphone experiments show that the proposed method is more robust to measurement noises than the existing calibration method.

### 6.1. Synthetic Data

**Figure 5:**
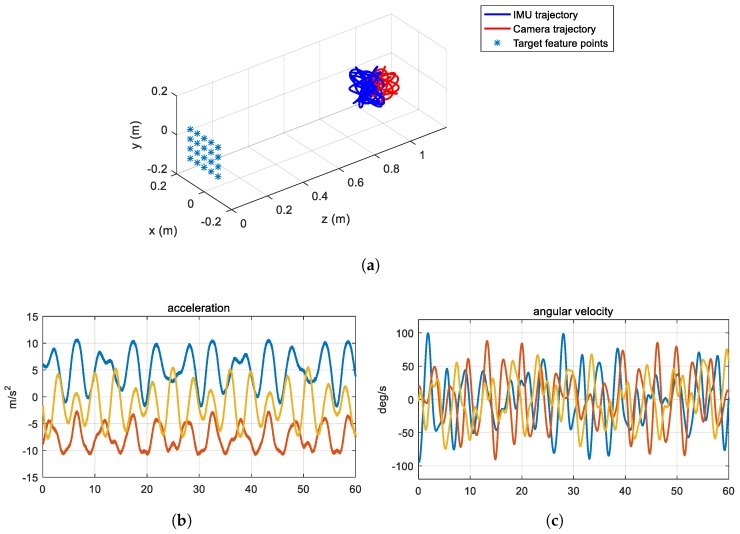
Synthetic data for the IMU–camera calibration: (**a**) trajectory of the rigidly connected IMU and camera; (**b**) synthesized acceleration measurements; and (**c**) synthesized angular velocity measurements. The blue, orange, and yellow color lines in (**b**,**c**) indicate the x-, y-, and z-axis measurements.

We generate the synthetic measurements for a frame length of 60 s. For this, we synthetically make 20 corners of a checkerboard pattern in the world coordinate and a smooth trajectory of the mobile platform by applying a sinusoidal function, as shown in Figure 5a. The points in the world coordinate are projected to the image coordinate as in [40], and are used as synthetic visual measurements for IMU–camera calibration. We set the image resolution to 1280 × 960. The focal length is set to 700 and the radial distortions are set to [0.1 −0.1]${}^{⊤}$. The rolling shutter readout time $\lambda_{rs}$ is set to 41.8 μs. The standard deviation of Gaussian noises for the visual measurements is set to 1 pixel. The IMU acceleration and angular velocity measurements along the world coordinate are obtained by differentiating the trajectory of the IMU. Then, to generate uncalibrated measurements, intrinsic parameters including a scale factor, a misalignment, a bias, and gravity in the world coordinate are used to perform the inertial measurements based on Equation (12). The scale factors ($s_{a}$, $s_{g}$), misalignments ($m_{a}$, $m_{g}$), and biases ($b_{a}$, $b_{g}$) are set to 1.1, 1.1, 0.03, 0.03, 0.1, and 0.1, respectively. These parameters are represented by a 3D vector and the elements of each vector are set to the same value. For instance, $m_{a}={\left[0.03,0.03,0.03\right]}^{⊤}$. Finally, we apply additive white Gaussian noise to the inertial measurements. Figure 5b,c shows an example of the synthetic inertial measurements. The frame rates of the IMU and the camera measurements are set to 100 and 25 Hz, respectively. For the extrinsic parameters, the relative rotation, relative translation, and time delay between the IMU and the camera are set to [50 −50 50]${}^{\circ }$, [50 50 −50]${}^{⊤}$ mm, and 100 ms, respectively. The calibration parameters are initialized as described in Section 5.3.

**Figure 6:**
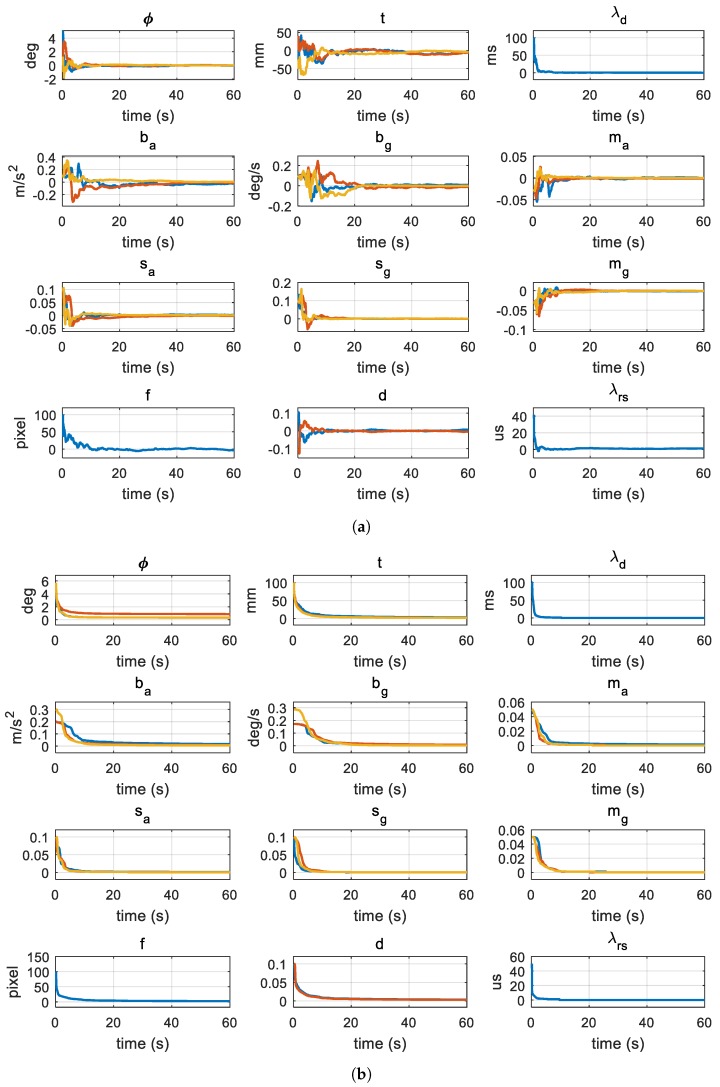
State errors and their covariances: The state contains calibration parameters, which are estimated by the filter of the proposed method on the synthetic data. The noise parameter is set to the mean of the estimates by the proposed method ($\sigma_{a}=0.1$ m/s${}^{2}$/$\sqrt{\mathrm{Hz}}$, $\sigma_{g}=0.1$ deg/s/$\sqrt{\mathrm{Hz}}$). The blue, orange, and yellow color lines of the 3-dimensional states and covariances (ϕ, t, $b_{a}$, $b_{g}$, $s_{a}$, $s_{g}$, $m_{a}$, and $m_{g}$) mean the x-, y-, and z-axis estimates. The blue and orange color lines of the 2-dimensional state and covariance d mean $d_{1}$, $d_{2}$. The 1-dimensional states and covariances ($\lambda_{d}$, $\lambda_{rs}$, and *f*) are represented by the blue color lines: (**a**) state error; and (**b**) state error covariance.

In the first experiment, we evaluate the proposed method with fixed IMU noise parameters, which represent the noise density of the IMU set to $\sigma_{a}$ = 0.1 m/s${}^{2}$/$\sqrt{\mathrm{Hz}}$, and $\sigma_{g}$ = 0.1${}^{\circ }$/s/$\sqrt{\mathrm{Hz}}$. The purpose of this experiment is to show how accurately and stably the proposed method estimates the calibration and noise parameters. Figure 6 shows the estimates of the calibration parameters and the standard deviation of their errors, which are a square root of diagonals of the state error covariance, over the 60-s frame length in the calibration and noise identification step. In our framework, the calibration parameters and their covariances are estimated by the UKF while estimating the noise parameters using nonlinear optimization. The graphs in Figure 6 show the calibration estimates obtained by the UKF at optimal noise parameter estimates by nonlinear optimization. In Figure 6a, the errors of the states, including the rotation ${}_{C}^{I}R$, translation ${}_{C}^{I}t$, time delay $\lambda_{d}$, biases $b_{a}$ and $b_{g}$, misalignments $m_{a}$ and $m_{w}$, scale factors $s_{a}$ and $s_{w}$, and focal length *f*, the radial distortion d and the rolling shutter readout time $\lambda_{rs}$, are rapidly converged to zero. The errors are computed using the ground truth calibration parameters. The rotation errors are displayed in the Euler angle form for intuitive representation. In Figure 6b, the error standard deviations obtained by the UKF are rapidly converged.

Table 4 summarizes the final calibration and noise parameter estimates of the proposed method by running 100 Monte-Carlo simulations. We describe the mean and standard deviation of the estimates to show statistically meaningful results. Table 4d shows the noise parameter estimates obtained by the proposed method. We find that the ground truth of the noise parameter is within the error bound of the noise parameter estimates. This result indicates that the proposed noise identification reasonably estimates the noise parameters. Therefore, the estimated noise parameters can be used to further refine the calibration parameters in the refinement step. Table 4a–c shows the extrinsic and intrinsic parameter estimates of the IMU and the camera. The differences between the estimates and the ground truth are smaller than their standard deviations. This validates that the proposed method accurately estimates all calibration parameters and noise parameters.

In the second experiment, we generate 100 sequences based on different IMU noise parameters, and, using these sequences, we analyze the proposed calibration method with and without the noise density estimation. The purpose of this experiment is to show that how the proposed noise identification robustly estimates the calibration parameters under unknown IMU noise density. The noise parameters $\sigma_{a},\sigma_{w}$ are randomly selected from 0.001 to 0.2, and the generated noises are added to the inertial measurements. These values cover most of the noise characteristics from low- to high-cost IMUs. We run the proposed method with noise estimation and fixed noise parameters ($\sigma_{a}/\sigma_{w}$ = 0.2/0.2 and 0.001/0.001). Table 5 shows the calibration parameter estimates of the proposed method with and without noise estimation. With noise estimation, the proposed calibration method estimates accurate calibration parameters. The mean of errors between the estimated results and the ground truth is smaller than the standard deviation of the estimates. On the contrary, fixed noise densities cause inaccurate and unstable estimates. The mean of the translation estimates without noise estimation is out of the error bound, and their standard deviation is about two times larger than that of the proposed method with noise estimation. For bias estimates, the mean of the estimates is not within the error bound and their standard deviation is larger. Besides, the estimates of the focal length and rolling shutter readout time are largely affected by the IMU noise parameters, whereas the rotation, misalignments, scale factors, and radial distortions are not dependent on the IMU noise parameters. These results validate that the proposed noise identification approach improves the accuracy and stability of the calibration parameter estimation, especially under unknown IMU noise density.

### 6.2. Real Data

For the real-data experiments, we use two setups: a self-designed system and a commercial smartphone. Since it is difficult to obtain ground truth for real data, we indirectly evaluate the performance of the proposed method using the results of existing methods and the hand-measured values. In addition, we use the standard deviation of estimates as an evaluation metric to analyze the algorithm stability. Moreover, the loop closing error metric with two cameras and the IMU is utilized to evaluate extrinsic calibration performance.

**Figure 7:**
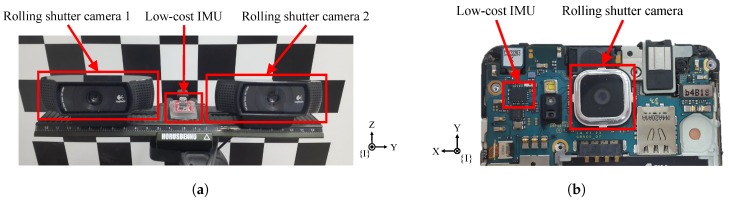
Two types of the low-cost IMU and the rolling shutter camera setup for real-data experiments: (**a**) our self-designed system having two rolling shutter cameras and a low-cost IMU; and (**b**) Samsung Galaxy Alpha (Samsung, Seoul, South Korea) having a rolling shutter camera and a low-cost IMU.

At first, we evaluate the proposed method using the self-designed system, which consists of two rolling shutter cameras and a low-cost IMU. Figure 7a shows our experimental setup. The rolling shutter cameras are Logitech C80 (Logitech, Lausanne, Switzerland) and the low-cost IMU is Withrobot myAHRS+. The image resolution is 640 × 480, and the frame rates of the camera and the IMU are, respectively, 30 and 100 Hz. We record a set of measurements for approximately 90 s and perform experiments on the 10 datasets.

Table 6 demonstrates calibration parameter estimates of camera 1 and the IMU. For extrinsic and IMU intrinsic parameters, we compare our method with the existing calibration method (i.e., the Kalibr-imucam method [19]) and the hand-measured values. The noise density of the IMU for the Kalibr-imucam method is set to the noise density estimated by the proposed method. Table 6a describes the extrinsic parameter estimates of the proposed method, the Kalibr-imucam method, and the hand-measured values. The rotation parameter ${}_{C}^{I}q$ is parameterized by Euler angle ϕ instead of unit quaternion for intuitive understanding. The average rotation, translation, and time delay estimates of the proposed and Kalibr-imucam methods are close to each other, and the average rotation and translation estimates of both methods are within the error bound of the hand-measured value. In addition, the standard deviation of the extrinsic parameter estimates from both methods is very small. This comparison shows that the proposed method successfully estimates the extrinsic parameters; moreover, the estimated noise parameters from the proposed method lead to the successful calibration of the existing calibration method.

Furthermore, with the two rolling shutter cameras, we evaluate the extrinsic calibration performance using the loop-closing error metric, which is defined as the transformation error $\delta T={}_{C_{1}}^{I}T\;\;{}_{C_{2}}^{C_{1}}T\;\;{{}_{C_{2}}^{I}T}^{-1}$ between the low-cost IMU and the two cameras (i.e., $C_{1}$ and $C_{2}$). Here, ${}_{C_{1}}^{I}T$ represents the relative transformation from the IMU to camera 1, ${}_{C_{2}}^{C_{1}}T$ represents the relative transformation between the two cameras, and ${}_{I}^{C_{2}}T$ denotes the relative transformation from camera 2 to the IMU. ${}_{C_{2}}^{C_{1}}T$ is estimated through the stereo camera calibration algorithm [43], and ${}_{C_{1}}^{I}T$ and ${}_{C_{2}}^{I}T^{-1}$ are estimated through the IMU–camera calibration algorithm. We run 10 sequences with the left camera–IMU and right camera–IMU. Then, we compute 10 × 10 loop closing errors by using bipartite matching (i.e., total 100 sets). Table 6b shows the mean and standard deviation of the loop closing errors on rotation and translation motions. The proposed and Kalibr-imucam methods produce very small errors on rotation and translation. Since the error bound of ${}_{C_{2}}^{C_{1}}T$ is about ±2 mm, the errors in our method and the Kalibr-imucam method are negligible.

Table 6c describes the IMU intrinsic parameter estimates of the proposed and Kalibr-imucam methods. We do not compare classic IMU intrinsic calibration methods [7,8,9] because they require special equipment such as robot arms to estimate intrinsic parameters. Besides, since the Kalibr-imucam method regards biases as time-varying parameters, unlike the proposed method, we do not compare it in this table. In both methods, the standard deviations of the misalignments and the scale factors are small enough, which indicates that the estimates are reliable. The results show that the *z*-axis bias of the accelerometer is larger than the *x*,*y*-axis biases due to the effect of the gravity vector located on the *z*-axis of the IMU coordinate (i.e., *z*-axis values of inertial measurements are much larger than those of *x*,*y*-axis). Interestingly, the misalignments are close to zero and the scale factors are close to one. This means that the IMU is somewhat intrinsically calibrated, although it is a low-cost sensor. The average estimates of the misalignments and scale factors from both methods have small differences because the Kalibr-imucam method also considers the effects of linear accelerations on gyroscopes. However, there are negligible numerical errors in the misalignments and scale factors.

For camera intrinsic parameters, we compare our method with the Kalibr-rs method [12] and the MATLAB calibration toolbox, as shown in Table 6d. The Kalibr-rs method uses image sequences, which are the same measurements as those used for the proposed method, and the MATLAB toolbox uses 20 still images of the same checker board. The focal length and radial distortion estimates of the three methods are close to each other. In addition, the average rolling shutter readout time estimates of the proposed method are close to the average estimates of the Kalibr-rs method. Although there are small differences between the estimates obtained from the three methods, they are negligible. Besides, the smaller standard deviation of the estimates from the proposed method indicates that the proposed method is more reliable than the Kalibr-rs method. This comparison of the intrinsic parameter estimates from the proposed and the two existing methods, which use various measurements, validates the camera intrinsic calibration of the proposed method.

Table 6e describes the estimated noise parameters. The standard deviation of IMU noise density estimates is small enough and their mean can be used for other sensor fusion algorithms such as IMU–camera calibration and visual-inertial SLAM methods. Although we do not compare the noise density provided from the other algorithms or manufacturers, the noise estimation performance of the proposed method is indirectly verified through comparison with extrinsic parameters of the hand-measured value and camera intrinsic parameters from the MATLAB Toolbox. In addition, the successful calibration of the Kalibr-imucam method with the noise density estimates obtained from the proposed method validates the noise estimation of the proposed method.

Second, we evaluate the proposed method using a smartphone “Samsung Galaxy Alpha”, which is equipped with a rolling shutter camera and a low-cost IMU; its configuration is shown in Figure 7b. The image resolution is 720 × 480, and the frame rates of the camera and the IMU are, respectively, 30 and 50 Hz. We record 10 sets of measurements whose frame length is about 90 s.

Table 7 shows the calibration parameter estimates of the smartphone. We compare the proposed method with the existing calibration method (i.e., Kalibr-imucam [19]) and hand-measured values, similarly to the above experiment. The average rotation estimates of the proposed method and the Kalibr-imucam method are close to those of the hand-measured value. The standard deviation of the rotation estimates from the proposed method is smaller than that obtained from the Kalibr-imucam method, and this result indicates that the proposed method is more reliable than the Kalibr-imucam method. Besides, the translation estimates of the Kalibr-imucam method are converged to unrealistic values that are zero, but our estimates are close to the hand-measured value. The average time delay estimates of the proposed method are close to those of [19]; however, the standard deviation is large because an irregular time delay occurs due to the camera module activation of the smartphone. In summary, in this experiment, the extrinsic calibration of the proposed method outperforms the Kalibri-imucam method unlike the first experiment, which uses the self-designed setup. We argue that the proposed method is more robust than the noisy measurements because our framework is based on the graybox method, which internally uses the Kalman filter. In practice, the rolling shutter camera and the IMU of the commercial smartphone contain larger noises than those of the self-designed setup, as described in Table 7d.

Table 7d demonstrates the IMU intrinsic estimates of the proposed method. Similar to the results of the first experiment, the *z*-axis bias of the accelerometer is larger than those of the other axes. The misalignment and scale factor estimates of the accelerometer from the proposed and Kalibri-imucam methods are, respectively, close to zero and one, and the biases and misalignments of the gyroscope are close to zero. This result indicates that they were already intrinsically calibrated. However, the scale factors of the gyroscope are calibrated through the proposed and Kalibr-imucam methods, as shown in the table. Although the scale factor estimates from two methods are slightly different, the low standard deviation of our estimates indicates that the proposed method is more reliable.

For camera intrinsic parameters, we compare our estimates to the Kalibr-rs method [12] and the MATLAB calibration toolbox, as shown in Table 7c. The focal length, radial distortion, and rolling shutter readout time estimates of the proposed method are close to the estimates of two existing methods. Although the estimates of the second coefficient of radial distortion are different for each method, the first coefficient is more important than the second. Besides, the proposed method provides the lower standard deviation of the rolling shutter readout time estimates, as in the first experiment.

Table 7d describes the estimated noise densities. They are two times larger than the noise density estimated on the smartphone setup. This means that the IMU noise of the smartphone setup is more severe than that of the self-designed setup. This experiment shows that a full calibration of off-the-shelf devices is possible with the proposed method.

Furthermore, we compare the operating time of the existing method (Kalibr [12,19]) and our method. The experimental environment for the comparison is Intel i7-4790K CPU running at a single core 4.00 GHz. Table 8 demonstrates the operating time of the Kalibri [12,19] and the proposed method. The timing statistics are measured on the 10 datasets used for the above “Samsung Galaxy Alpha” experiment. In case of the existing method, it is required to use both the Kalibr-rs [12] and the Kalibr-imucam [19] together because they separately estimate the intrinsic and extrinsic parameters, whereas, the proposed method does not. The total operating time of the Kalibr method is about 2 h in average for the full calibration. In particular, the operating time of Kalibri-rs [12] is longer than 1 h because of the iterative batch optimization for adaptive knot placement. However, the proposed method estimates noise parameters as well as calibration parameters, and it is 1.733 times faster than the Kalibr method in average.

**Figure 8:**
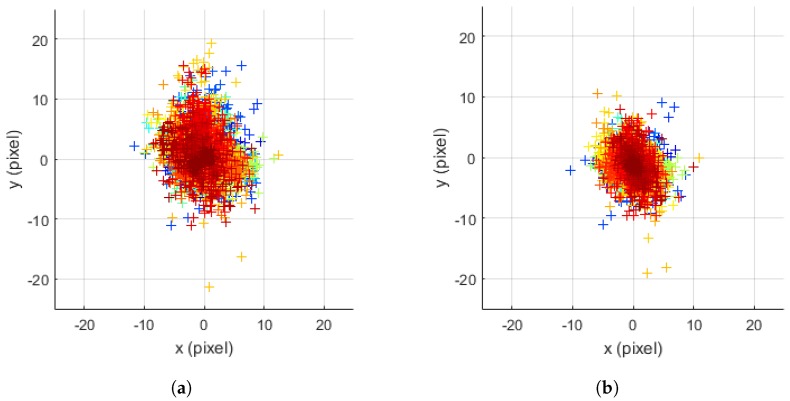
Prediction errors in the UKF for motion estimation with the calibration parameters of the Kalibr and the proposed method. The prediction errors of visual measurements (corners) for all frames are recorded, and a cross represents a prediction error. The color of crosses means the index of a frame (i.e., blue indicates the start of the sequence and red does the end of the sequences): (**a**) Kalibr-imucam [19] and Kalibr-rs [12] methods, with average RMSE of 3.03 pixel; and (**b**) proposed method, with average RMSE of 2.45 pixel.

We also compare the prediction errors of the UKF for motion estimation with calibration parameters estimated by the Kalibr method and the proposed method. In this experiment, the states of the UKF are a position, velocity, and orientation of the IMU, and they are predicted by inertial measurements and corrected by visual measurements, which are corners of a checkerboard pattern. We record a set of inertial and visual measurements using the smartphone “Samsung Galaxy Alpha” for 60 s. The prediction errors using the Kalibr are accumulated due to the IMU noise and they result in the motion drift. However, Figure 8 shows that the calibration parameters of the proposed method reduce the mean and variance of prediction errors. The average RMSE in terms of pixel for all frames decreases from 3.03 to 2.45 (about 19.2%).

**Figure 9:**
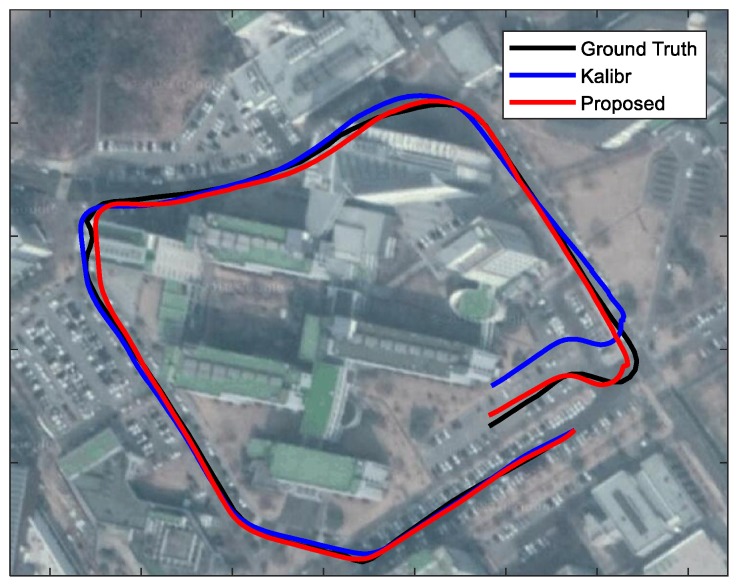
Outdoor localization results with the calibration parameters of the Kalibr and the proposed method. The estimated trajectories are overlaid on Google Map. The visual and inertial measurements are collected by the smartphone “Samsung Galaxy Alpha” under vehicle driving condition. The length of the whole trajectory is about 0.8 km. The ground truth trajectory is obtained from GPS measurements.

Finally, we compare the localization accuracy with calibration parameters estimated by the Kalibr method and the proposed method. In this experiment, we capture image sequences and inertial measurements using the smartphone “Samsung Galaxy Alpha” in an outdoor vehicle driving environment. The trajectories are estimated by VINS-mono [44], which is an open source visual-inertial SLAM algorithm. In this experiment, we do not use loop closure correction and online calibration on the extrinsic parameter and temporal delay. Figure 9 shows the estimated trajectory with the proposed method is close to the ground truth trajectory, whereas the trajectory estimates with the Kalibr method suffers from scale drift as the trajectory becomes longer. The experimental results validate that the proposed method improves the performance of the real-world system as well.

## 7. Conclusions

This paper proposes a robust and accurate calibration method for a low-cost IMU and a rolling shutter camera. The proposed joint calibration estimates not only the intrinsic and extrinsic parameters but also the IMU noise parameters. To improve calibration efficiency including runtime, we divide the framework into two steps. In the first step, we roughly estimate the intrinsic and extrinsic parameters through filtering while estimating the noise parameters in the optimization module. In the second step, we refine the intrinsic and extrinsic parameters via the optimization module while estimating the sensor motion in filtering. The experimental results of the synthetic data demonstrate the superiority of our framework, and, in particular, the experiments on two real-data setups validate the performance of the proposed method in off-the-shelf devices.

As a result, the proposed method enhances the runtime about 73.3% and reduces IMU drift by 19.2% in comparison with those of the Kalibr method [12,19]. In particular, the results of the visual-inertial SLAM on the real-world system demonstrates that the proposed method outperforms the Kalibr method.

## Figures and Tables

**Table 1:** Comparison of related studies: the parameters denote IMU–camera translation (${}_{C}^{I}t$), IMU–camera rotation (${}_{C}^{I}q$), IMU–camera time delay ($\lambda_{t}$), translation between IMU axes (${}_{I}^{B}t$), camera focal length (*f*), camera radial distortion (d), rolling shutter readout time ($\lambda_{rs}$), IMU bias ($b_{a,g}$), IMU scale factor($s_{a,g}$), IMU misalignment($m_{a,g}$), and IMU noise density ($\sigma_{a,g}$). Refer to Figure 2 and Table 2 for the notations *I*, *C*, and *W*.

	Estimator	Extrinsics			Camera Intrinsics		IMU Intrinsics			IMU Noise Densities
		${}_{C}^{I}t$, ${}_{C}^{I}q$	$\lambda_{t}$	${}_{I}^{B}t$	*f*, d	$\lambda_{\mathrm{rs}}$	$b_{a,g}$	$s_{a,g}$	$m_{a,g}$	$\sigma_{a,g}$
Zachariah [16]	UKF	√					√	√	√	
Lee [18]	UKF	√					√	√	√	
Rehder [19]	LM	√	√	√			√	√	√	
Li [17]	MSCKF	√	√		√	√	√	√	√	
Proposed	Graybox	√	√		√	√	√	√	√	√

**Table 2:** The parameter description.

	Notation	Description	Dimension	Unit
Extrinsic parameters	${}_{C}^{I}t$	Translation between IMU and camera	3	mm
	${}_{C}^{I}q$	Rotation from IMU to camera (unit-quaternion)	4	-
	$\lambda_{d}$	Time delay between IMU and camera measurements	1	ms
IMU intrinsic parameters	$b_{a}$	Bias of acceleration measurements	3	m/s${}^{2}$
	$b_{g}$	Bias of gyroscope measurements	3	rad/s
	$m_{a}$	Misalignment of accelerometer axes	3	-
	$m_{g}$	Misalignment of gyroscope axes	3	-
	$s_{a}$	Scale factors of accelerometer axes	3	-
	$s_{g}$	Scale factors of gyroscope axes	3	-
Camera intrinsic parameters	*f*	Focal length of camera	1	pixel
	d	Radial distortion coefficients	2	-
	$\lambda_{rs}$	Rolling shutter readout time	1	μs
Noise parameters	$\sigma_{a}$	Noise density of accelerometer	1	m/s${}^{2}/\sqrt{\mathrm{Hz}}$
	$\sigma_{g}$	Noise density of gyroscope	1	rad/s/$\sqrt{\mathrm{Hz}}$

**Table 3:** Initial state error covariance. The elements in a variable are set to equal values.

$P_{{}_{I}^{W}p}$	$P_{{}_{I}^{W}v}$	$P_{{}_{I}^{W}q}$	$P_{{}_{C}^{I}t}$	$P_{{}_{C}^{I}q}$	$P_{\lambda_{d}}$	$P_{b_{a}}$	$P_{b_{g}}$	$P_{m_{a}}$	$P_{m_{g}}$	$P_{s_{a}}$	$P_{s_{g}}$	$P_{f}$	$P_{d}$	$P_{\lambda_{\mathrm{rs}}}$
$0.05^{2}$	$0.05^{2}$	$0.05^{2}$	$100^{2}$	$0.05^{2}$	$100^{2}$	$0.2^{2}$	$0.003^{2}$	$0.05^{2}$	$0.05^{2}$	$0.1^{2}$	$0.1^{2}$	$10^{2}$	$0.1^{2}$	$0.05^{2}$

**Table:** (**a**)

	$\phi_{x}$ [deg]	$\phi_{y}$ [deg]	$\phi_{z}$ [deg]	$t_{x}$ [mm]	$t_{y}$ [mm]	$t_{z}$ [mm]	$\lambda_{d}$ [ms]
Estimates	50.0 ± 0.0	50.0 ± 0.0	50.0 ± 0.0	48.3 ± 4.6	48.7 ± 3.2	−49.0 ± 2.8	100.1 ± 0.1
Ground truth	50.0	−50.0	50.0	50.0	50.0	50.0	100.0

**Table:** (**b**)

	$b_{a,x}$ **[m/s${}^{2}$]**	$b_{a,y}$ **[m/s${}^{2}$]**	$b_{a,z}$ **[m/s${}^{2}$]**	$b_{g,x}$ **[deg/s]**	$b_{g,y}$ **[deg/s]**	$b_{g,z}$ **[deg/s]**
Estimates	0.11 ± 0.02	0.10 ± 0.02	0.10 ± 0.01	0.10 ± 0.01	0.09 ± 0.01	0.10 ± 0.00
Ground truth	0.10	0.10	0.10	0.10	0.10	0.10
	$m_{a,x}$ **[−]**	$m_{a,y}$ **[−]**	$m_{a,z}$ **[−]**	$m_{g,x}$ **[−]**	$m_{g,y}$ **[−]**	$m_{g,z}$ **[−]**
Estimates	0.031 ± 0.003	0.029 ± 0.001	0.030 ± 0.001	0.030 ± 0.001	0.029 ± 0.001	0.030 ± 0.001
Ground truth	0.030	0.030	0.030	0.030	0.030	0.030
	$s_{a,x}$ **[−]**	$s_{a,y}$ **[−]**	$s_{a,z}$ **[−]**	$s_{g,x}$ **[−]**	$s_{g,y}$ **[−]**	$s_{g,z}$ **[−]**
Estimates	1.098 ± 0.002	1.100 ± 0.001	1.099 ± 0.001	1.100 ± 0.001	1.100 ± 0.001	1.099 ± 0.001
Ground truth	1.100	1.100	1.100	1.100	1.100	1.100

**Table:** (**c**)

	*f* [pixel]	$d_{1}$ [−]	$d_{2}$ [−]	$\lambda_{\mathrm{rs}}$ [μs]
Estimates	699.6 ± 1.8	0.10 ± 0.00	−0.10 ± 0.00	41.7 ± 0.1
Ground truth	700.0	0.10	−0.10	41.8

**Table:** (**d**)

	$\sigma_{a}$ [m/s${}^{2}$/$\sqrt{\mathrm{Hz}}$]	$\sigma_{g}$ [deg/s/$\sqrt{\mathrm{Hz}}$]
Estimates	0.10 ± 0.02	0.10 ± 0.15
Ground Truth	0.10	0.10

**Table:** (**a**)

	$\sigma_{a}$/$\sigma_{g}$	$\phi_{x}$ [deg]	$\phi_{y}$ [deg]	$\phi_{z}$ [deg]	$t_{x}$ [mm]	$t_{y}$ [mm]	$t_{z}$ [mm]	$\lambda_{d}$ [ms]
Estimates	0.2/0.2	−50.0 ± 0.2	50.1 ± 0.1	−49.9 ± 0.2	37.2 ± 9.3	46.9 ± 6.7	−26.9 ± 7.0	98.6 ± 2.2
	0.001/0.001	−50.0 ± 0.2	50.0 ± 0.1	−50.0 ± 0.2	48.2 ± 12.1	48.0 ± 7.6	−47.9 ± 7.7	99.5 ± 2.1
	auto	−50.0 ± 0.1	50.0 ± 0.0	−50.0 ± 0.1	50.9 ± 5.1	52.0 ± 3.4	−48.6 ± 4.0	100.0 ± 0.9
Ground truth	-	50.0	−50.0	50.0	50.0	50.0	50.0	100.0

**Table:** (**b**)

	**$\sigma_{a}$/$\sigma_{g}$**	**$b_{a,x}$ [m/s${}^{2}$]**	**$b_{a,y}$ [m/s${}^{2}$]**	**$b_{a,z}$ [m/s${}^{2}$]**	**$b_{g,x}$ [deg/s]**	**$b_{g,y}$ [deg/s]**	**$b_{g,z}$ [deg/s]**
Estimates	0.2/0.2	0.01 ± 0.00	0.04 ± 0.02	0.07 ± 0.02	0.03 ± 0.03	0.08 ± 0.02	0.06 ± 0.02
	0.001/0.001	0.10 ± 0.06	0.10 ± 0.03	0.10 ± 0.02	0.10 ± 0.01	0.10 ± 0.02	0.10 ± 0.00
	auto	0.10 ± 0.04	0.10 ± 0.02	0.10 ± 0.01	0.09 ± 0.01	0.10 ± 0.01	0.10 ± 0.01
Ground truth	-	0.1	0.1	0.1	0.10	0.10	0.10
	**$\sigma_{a}$/$\sigma_{g}$**	$m_{a,x}$ **[−]**	$m_{a,y}$ **[−]**	$m_{a,z}$ **[−]**	$m_{g,x}$ **[−]**	$m_{g,y}$ **[−]**	$m_{g,z}$ **[−]**
Estimates	0.2/0.2	0.021 ± 0.002	0.023 ± 0.002	0.032 ± 0.02	0.028 ± 0.002	0.030 ± 0.002	0.028 ± 0.002
	0.001/0.001	0.030 ± 0.005	0.029 ± 0.002	0.030 ± 0.002	0.030 ± 0.002	0.029 ± 0.001	0.030 ± 0.001
	auto	0.030 ± 0.004	0.028 ± 0.001	0.030 ± 0.001	0.030 ± 0.001	0.029 ± 0.001	0.030 ± 0.001
Ground truth	-	0.030	0.030	0.030	0.030	0.030	0.030
	**$\sigma_{a}$/$\sigma_{g}$**	$s_{a,x}$ **[−]**	$s_{a,y}$ **[−]**	$s_{a,z}$ **[−]**	$s_{g,x}$ **[−]**	$s_{g,y}$ **[−]**	$s_{g,z}$ **[−]**
Estimates	0.2/0.2	1.104 ± 0.002	1.092 ± 0.002	1.096 ± 0.003	1.098 ± 0.002	1.099 ± 0.001	1.096 ± 0.003
	0.001/0.001	1.100 ± 0.005	1.100 ± 0.002	1.100 ± 0.002	1.100 ± 0.001	1.100 ± 0.001	1.100 ± 0.002
	auto	1.099 ± 0.002	1.100 ± 0.002	1.100 ± 0.002	1.100 ± 0.001	1.100 ± 0.001	1.100 ± 0.001
Ground truth	-	1.100	1.100	1.100	1.100	1.100	1.100

**Table:** (**c**)

	$\sigma_{a}$/$\sigma_{g}$	*f* [pixel]	$d_{1}$ [−]	$d_{2}$ [−]	$\lambda_{\mathrm{rs}}$ [μs]
Estimates	0.2/0.2	704.9 ± 2.8	0.11 ± 0.01	−0.11 ± 0.01	41.1 ± 0.5
	0.001/0.001	697.1 ± 9.6	0.09 ± 0.01	0.09 ± 0.01	39.3 ± 3.1
	auto	701.4 ± 2.9	0.10 ± 0.01	−0.10 ± 0.01	41.3 ± 0.7
Ground truth	-	700.0	0.10	−0.10	41.8

**Table:** (**a**)

	$\phi_{x}$ [deg]	$\phi_{y}$ [deg]	$\phi_{z}$ [deg]	$t_{x}$ [mm]	$t_{y}$ [mm]	$t_{z}$ [mm]	$\lambda_{d}$ [ms]
Proposed	−88.9 ± 0.1	1.0 ± 0.0	271.1 ± 0.1	24.5 ± 1.4	−70.1 ± 0.8	20.0 ± 1.1	−21.3 ± 0.8
Kalibr-imucam [19]	−89.4 ± 0.0	1.2 ± 0.1	270.7 ± 0.1	22.3 ± 1.1	−70.7 ± 0.7	19.9 ± 0.9	−24.1 ± 0.5
Hand	−90.0 ± 2.5	0.0 ± 2.5	270.0 ± 2.5	25.0 ± 5.0	−70.0 ± 5.0	20.0 ± 5.0	-

**Table:** (**b**)

	$\phi_{x}$ [deg]	$\phi_{y}$ [deg]	$\phi_{z}$ [deg]	$t_{x}$ [mm]	$t_{y}$ [mm]	$t_{z}$ [mm]
Proposed	−0.8 ± 0.1	−0.6 ± 0.1	−0.2 ± 0.0	2.9 ± 2.2	1.6 ± 1.9	−1.5 ± 2.0
Kalibr-imucam [19]	−0.8 ± 0.1	0.5 ± 0.1	0.1 ± 0.1	−2.5 ± 1.1	−1.2 ± 2.1	2.3 ± 1.5

**Table:** (**c**)

	**$b_{a,x}$ [m/s${}^{2}$]**	**$b_{a,y}$ [m/s${}^{2}$]**	**$b_{a,z}$ [m/s${}^{2}$]**	**$b_{g,x}$ [deg/s]**	**$b_{g,y}$ [deg/s]**	**$b_{g,z}$ [deg/s]**
Proposed	−0.02 ± 0.01	−0.03 ± 0.01	−0.13 ± 0.01	−0.13 ± 0.02	0.05 ± 0.02	−0.04 ± 0.02
	$m_{a,x}$ **[−]**	$m_{a,y}$ **[−]**	$m_{a,z}$ **[−]**	$m_{g,x}$ **[−]**	$m_{g,y}$ **[−]**	$m_{g,z}$ **[−]**
Proposed	0.000 ± 0.001	−0.004 ± 0.001	0.000 ± 0.001	−0.002 ± 0.001	0.002 ± 0.001	0.001 ± 0.001
Kalibr-imucam [19]	0.002 ± 0.001	0.003 ± 0.001	0.000 ± 0.001	−0.002 ± 0.001	0.000 ± 0.001	−0.002 ± 0.001
	$s_{a,x}$ **[−]**	$s_{a,y}$ **[−]**	$s_{a,z}$ **[−]**	$s_{g,x}$ **[−]**	$s_{g,y}$ [−]	$s_{g,z}$ **[−]**
Proposed	1.001 ± 0.001	1.002 ± 0.001	1.007 ± 0.002	1.003 ± 0.002	1.001 ± 0.002	0.997 ± 0.003
Kalibr-imucam [19]	0.999 ± 0.000	0.999 ± 0.000	1.002 ± 0.000	1.000 ± 0.000	0.999 ± 0.001	0.999 ± 0.001

**Table:** (**d**)

	*f* [pixel]	$d_{1}$ [−]	$d_{2}$ [−]	$\lambda_{\mathrm{rs}}$ [μs]
Proposed	616.0 ± 1.4	0.08 ± 0.01	−0.14 ± 0.03	70.1 ± 1.3
Kalibr-rs [12]	623.4 ± 3.6	0.08 ± 0.01	−0.07 ± 0.08	60.7 ± 7.9
MATLAB Toolbox	611.1	0.10	−0.17	–

**Table:** (**e**)

	$\sigma_{a}$ [m/s${}^{2}$/$\sqrt{\mathrm{Hz}}$]	$\sigma_{g}$ [deg/s/$\sqrt{\mathrm{Hz}}$]
Proposed	0.028 ± 0.004	0.089 ± 0.011

**Table:** (**a**)

	$\phi_{x}$ [deg]	$\phi_{y}$ [deg]	$\phi_{z}$ [deg]	$t_{x}$ [mm]	$t_{y}$ [mm]	$t_{z}$ [mm]	$\lambda_{d}$ [ms]
Proposed	−89.6 ± 0.1	−0.1 ± 0.1	180.5 ± 0.1	−18.5 ± 2.4	−2.0 ± 2.6	1.4 ± 2.2	−39.2 ± 29.1
Kalibr-imucam [19]	−89.8 ± 0.3	−0.7 ± 0.2	180.2 ± 0.3	0.0 ± 0.0	0.0 ± 0.1	0.0 ± 0.0	−30.0 ± 30.2
Hand	−90.0 ± 2.5	0.0 ± 2.5	180.0 ± 2.5	−20.0 ± 5.0	0.0 ± 5.0	0.0 ± 5.0	-

**Table:** (**b**)

	**$b_{a,x}$ [m/s${}^{2}$]**	**$b_{a,y}$ [m/s${}^{2}$]**	**$b_{a,z}$ [m/s${}^{2}$]**	**$b_{g,x}$ [deg/s]**	**$b_{g,y}$ [deg/s]**	**$b_{g,z}$ [deg/s]**
Proposed	−0.06 ± 0.02	0.03 ± 0.04	−0.11 ± 0.02	0.01 ± 0.0	0.01 ± 0.04	−0.01 ± 0.04
	$m_{a,x}$ **[−]**	$m_{a,y}$ **[−]**	$m_{a,z}$ **[−]**	$m_{g,x}$ **[−]**	$m_{g,y}$ **[−]**	$m_{g,z}$ **[−]**
Proposed	0.002 ± 0.001	0.004 ± 0.001	0.000 ± 0.001	0.000 ± 0.002	−0.001 ± 0.002	0.000 ± 0.002
Kalibr-imucam [19]	0.000 ± 0.000	0.000 ± 0.000	0.000 ± 0.000	-0.003 ± 0.021	-0.001 ± 0.010	0.013 ± 0.013
	$s_{a,x}$ **[−]**	$s_{a,y}$ **[−]**	$s_{a,z}$ **[−]**	$s_{g,x}$ **[−]**	$s_{g,y}$ **[−]**	$s_{g,z}$ **[−]**
Proposed	1.008 ± 0.002	1.000 ± 0.002	1.009 ± 0.002	0.994 ± 0.002	1.003 ± 0.002	1.005 ± 0.004
Kalibr-imucam [19]	1.000 ± 0.000	1.000 ± 0.000	1.000 ± 0.000	0.965 ± 0.008	0.918 ± 0.015	0.987 ± 0.008

**Table:** (**c**)

	*f* [pixel]	$d_{1}$ [−]	$d_{2}$ [−]	$\lambda_{\mathrm{rs}}$ [μs]
Proposed	679.1 ± 2.6	0.18 ± 0.02	−0.30 ± −0.14	66.2 ± 0.4
Kalibr-rs [12]	686.0 ± 3.0	0.18 ± 0.01	−0.23 ± 0.12	61.1 ± 4.1
MATLAB Toolbox	683.9	0.22	−0.63	-

**Table:** (**d**)

	$\sigma_{a}$ [m/s${}^{2}$/$\sqrt{\mathrm{Hz}}$]	$\sigma_{g}$ [deg/s/$\sqrt{\mathrm{Hz}}$]
Proposed	0.057 ± 0.013	0.226 ± 0.117

**Table 8:** Timing comparison of the existing and proposed methods. Mean and standard deviation of the operating times are described together.

		Time (min)
Kalibri	Kalibri-imucam [19]	24.80 ± 2.89
	Kalibri-rs [12]	102.03 ± 42.94
	Total	126.83 ± 42.03
Proposed	Noise Parameter Initialization	24.62 ± 0.21
	Calibration & Noise Identification	27.18 ± 10.11
	Calibration Parameter Refinement	21.36 ± 12.10
	Total	73.16 ± 12.38
